# Mechanistic insights on age-related changes in heart-aorta-brain hemodynamic coupling using a pulse wave model of the entire circulatory system

**DOI:** 10.1152/ajpheart.00314.2023

**Published:** 2023-09-15

**Authors:** Arian Aghilinejad, Faisal Amlani, Sohrab P. Mazandarani, Kevin S. King, Niema M. Pahlevan

**Affiliations:** ^1^Department of Aerospace and Mechanical Engineering, University of Southern California, Los Angeles, California, United States; ^2^Laboratoire de Mécanique Paris-Saclay, Université Paris-Saclay, Paris, France; ^3^Department of Medicine, Keck School of Medicine, University of Southern California, Los Angeles, California, United States; ^4^Barrow Neurological Institute, Phoenix, Arizona, United States; ^5^Division of Cardiovascular Medicine, Department of Medicine, University of Southern California, Los Angeles, California, United States

**Keywords:** aging, arterial stiffening, hemodynamics, vascular dementia

## Abstract

Age-related changes in aortic biomechanics can impact the brain by reducing blood flow and increasing pulsatile energy transmission. Clinical studies have shown that impaired cardiac function in patients with heart failure is associated with cognitive impairment. Although previous studies have attempted to elucidate the complex relationship between age-associated aortic stiffening and pulsatility transmission to the cerebral network, they have not adequately addressed the effect of interactions between aortic stiffness and left ventricle (LV) contractility (neither on energy transmission nor on brain perfusion). In this study, we use a well-established and validated one-dimensional blood flow and pulse wave computational model of the circulatory system to address how age-related changes in cardiac function and vasculature affect the underlying mechanisms involved in the LV-aorta-brain hemodynamic coupling. Our results reveal how LV contractility affects pulsatile energy transmission to the brain, even with preserved cardiac output. Our model demonstrates the existence of an optimal heart rate (near the normal human heart rate) that minimizes pulsatile energy transmission to the brain at different contractility levels. Our findings further suggest that the reduction in cerebral blood flow at low levels of LV contractility is more prominent in the setting of age-related aortic stiffening. Maintaining optimal blood flow to the brain requires either an increase in contractility or an increase in heart rate. The former consistently leads to higher pulsatile power transmission, and the latter can either increase or decrease subsequent pulsatile power transmission to the brain.

**NEW & NOTEWORTHY** We investigated the impact of major aging mechanisms of the arterial system and cardiac function on brain hemodynamics. Our findings suggest that aging has a significant impact on heart-aorta-brain coupling through changes in both arterial stiffening and left ventricle (LV) contractility. Understanding the underlying physical mechanisms involved here can potentially be a key step for developing more effective therapeutic strategies that can mitigate the contributions of abnormal LV-arterial coupling toward neurodegenerative diseases and dementia.

## INTRODUCTION

The circulatory system operates based on a delicate hemodynamic balance between the heart, the aorta, and major target organs such as the brain (1, 2). In healthy young adults, interactions between the left ventricle (LV) and the aorta are optimized to guarantee the delivery of cardiac output (CO) with a modest pulsatile hemodynamic load on the LV (3, 4). In youth, the low impedance of a compliant aorta interacts with stiffer conduit arteries such as the carotid artery. This creates impedance mismatches and wave reflections at the aorta-brain boundaries that limit the transmission of excessive pulsatile energy into the cerebral microcirculation and protect the brain tissue (5, 6). It is worth noting that impedance mismatch is not the only theory explaining the brain’s protective mechanism against excessive pulsatile energy transmission. As highlighted in other studies, factors such as vessel size and area should also be considered (7, 8). What is unquestionable, however, is that alteration in wave dynamics due to aortic stiffening leads to increased pulsatile energy transmission to the brain, which can be detrimental to the cerebral microvasculature and brain tissues (8). The optimum hemodynamic coupling between the LV, the aorta, and the brain can be impaired because of age-related changes in aortic stiffness (2, 9). Indeed, stiffness increases with age and is one of the earliest pathological changes within the arterial wall, ultimately affecting the wave dynamics in the vasculature. This change can be identified before the onset of hypertension and may account for ethnic differences in cardiovascular and brain health (1, 10, 11). For heart-aorta coupling (LV arterial coupling), previous studies have shown that elevated aortic stiffness increases the LV pulsatile load, leading to an increase in LV mass which, in turn, contributes to the development of heart failure (HF) (12, 13). At the aorta-brain interface, it has been shown that disproportionate aortic stiffening increases aortic impedance, alters wave reflections, and increases the transmission of harmful pulsatile energy into the cerebrovascular network, ultimately leading to cognitive impairments such as Alzheimer’s and other related vascular dementia (14, 15).

Furthermore, population-based clinical studies have suggested that patients with HF with impaired LV function have worse degrees of cognitive impairment than age-matched individuals without HF (16, 17). HF has been proposed as a risk factor for Alzheimer’s disease (AD), where the current clinical hypothesis is that the decreased cerebral blood flow due to HF may contribute to the dysfunction of the neurovascular unit and hence may lead to impaired clearance of amyloid beta (17–20). In addition to the consequences of HF, age-associated changes in ventricular wall thickening and stiffening may trigger heart remodeling that can also affect cerebral hemodynamics. Although previous studies have attempted to elucidate the complex relationship between aortic stiffness and pulsatile energy transmission to the brain (3, 6, 21), these studies have not adequately addressed the effect of interactions between the aorta and the LV on such energy transmission (nor on brain perfusion). Indeed, recent work has focused only on aorta-brain coupling and has not studied the impact of cardiac dynamics on cerebral perfusion (14). This may be due to the inherent difficulties in studying the isolated effects of aortic wave dynamics and cardiac function on brain hemodynamics in clinical settings (22, 23).

The state of LV-aorta-brain coupling is mainly dominated by LV contractility (a major determinant of LV function), heart rate (a determinant of the fundamental frequency of propagated arterial waves), and aortic stiffness (a determinant of the buffering function of the aorta and pulse wave velocity). The optimal state of LV-aorta-brain coupling is achieved via the interplay of these three determinants (13, 21). The current study aims to gain mechanistic insight into age-related impacts on brain hemodynamics that are caused by alterations in the arterial system and cardiac function. In particular, we investigate the effects of LV contractility (as quantified by LV end-systolic elastance) and of aortic stiffness [as measured by pulse wave velocity (PWV)] on the transmission of pulsatile energy and flow to the brain. One-dimensional (1-D) arterial pulse wave models (based on axisymmetric Navier–Stokes formulations) are well established as physiologically relevant tools to study global cardiovascular function (24–26). In this work, we use such a modeling approach to the entire human circulation (27) using a high-order, fast Fourier transform (FFT)-based numerical methodology (28, 29).

## METHODS

### Physical Model of the Entire Human Circulation

**Figure 1.**
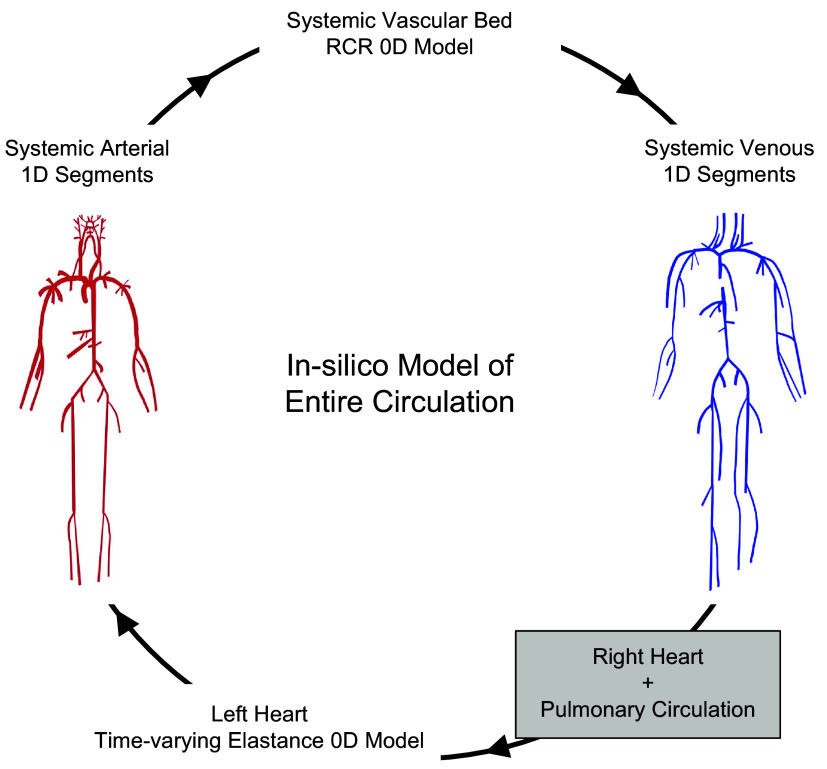
Closed-loop cardiovascular model consisting of 1-dimensional (1-D) segments coupled to 0-dimensional (0-D) lumped-parameter models of the heart and microvasculature.

A validated 1-D model (27) of the complete circulatory system, based on space-time variables, was used in this study. The physical model included 122 larger systemic arteries and 162 veins, each characterized by diameter, length, Young’s modulus, and wall thickness. Figure 1 illustrates the closed-loop cardiovascular model that consists of such 1-D segments for modeling wave propagation in larger arteries/veins, together with zero-dimensional (0-D) compartments for modeling all four heart chambers (including the left ventricle) as well as the (truncated) microvasculature. The arterial wall is assumed to be thin, incompressible, homogeneous, and isotropic. In this study, we focused on investigating the effect of LV dynamics and aortic stiffness on pulsatility transmission to the brain. Different levels of aortic rigidity were considered by using multiplicative factors of a minimum rigidity level $E_{1}\left(x\right)$ that corresponds to the baseline PWV (${\text{c}}_{0}$) that is initially prescribed in the model. To simulate different states of LV contractility (29–32), the end-systolic elastance ($E_{\text{es}}$) is varied. In this work, a value of $E_{\text{es}}$ = 2.5 mmHg/mL is considered as the control and normotensive case, whereas values below 1.5 mmHg/mL and larger than 3.5 mmHg/mL are considered to be of low and high contractility, respectively (33).

### Computational Model and Numerical Solver

We adopt a nonlinear and physiologically relevant fluid-structure model to simulate the complete circulation, particularly the different material properties encountered in various vascular segments (27, 34). For cross-sectional area A=A(x,t) and mean velocity over the cross section U = U(x,t) (yielding the flow rate as Q=AU), such a model can be expressed as a reduced-order nonlinear system for each vessel segment by the expression

(*1*) $$\left(\begin{array}{c} \frac{\partial A}{\partial \text{t}}\left(x,\;t\right)\\ \frac{\partial U}{\partial t}\left(x,\;t\right) \end{array}\right)=\;-\;\left(\begin{array}{c} \frac{\partial \left(AU\right)}{\partial x}\left(x,\;t\right)\\ U\frac{\partial U}{\partial x}\left(x,\;t\right)+\frac{1}{\rho }\frac{\partial \text{P}}{\partial x}\left(x,\;t\right)+\frac{2\left(\xi +2\right)\pi \mu U\left(x,\;t\right)}{\rho A\left(x,\;t\right)} \end{array}\right),$$

where ρ is a (constant) blood density, μ is a (constant) blood viscosity, and ξ is a given constant of an assumed axisymmetric velocity profile. The blood is assumed to be Newtonian (35, 36). The system is closed by an assumed elastic (tube law) that accounts for the fluid-structure interaction and can be given by the constitutive law (37).

(*2*) $$\text{P}={\text{P}}_{\text{ext}}+\frac{\beta \left(x\right)}{A_{\text{d}}}\left(\sqrt{A}-\sqrt{A_{\text{d}}}\right),\;\beta \left(x\right)=\frac{4}{3}\sqrt{\pi }E\left(x\right)h\left(x\right),$$

where ${\text{P}}_{\text{ext}}$ is the external and reference pressure, $A_{\text{d}}$ is the diastolic area, and β(x) is an expression of the arterial wall material properties in terms of elastic modulus E(x) (a measure of stiffness) and wall thickness h(x). To simulate multiple vessels, including vascular bifurcations or trifurcations, it is necessary to treat the fractal structure of the circulation network and, namely, branching points. These junctions effectively act as mathematical discontinuities in cross-sectional area and material properties. Physically, one must enforce continuity of total pressure and conservation of mass (flow rate) at junction points. For example, given a parent vessel p that splits into two daughter vessels $d_{\text{i}},\;i=1\text{,}2$, the corresponding mathematical conditions are given by

(*3*) $${\text{P}}_{\text{p}}+\frac{\rho }{2}U_{\text{p}}={\text{P}}_{d_{i}}+\frac{\rho }{2}U_{d_{i}},\;i=1,\;2,$$

(*4*) $$A_{\text{p}}U_{\text{p}}+A_{d_{1}}U_{d_{1}}+A_{d_{2}}U_{d_{2}}=0.$$

The overall numerical methodology for a vessel governed by *Eqs. 1** and **2*, together with the junction conditions of *Eqs. 3** and **4*, is provided by a high-order Fourier continuation approach for hemodynamics equations introduced by Amlani and Pahlevan (29). Such a methodology enables long-time and long-distance wave propagation with minimal numerical dispersion or diffusion errors (28, 29, 38). A brief description of the corresponding algorithm, as well as benchmark validation (37) of its implementation, is provided in appendix.

Following the works of Mynard and Smolich (27, 39), three types of vascular beds are considered: generic vascular beds (shown in Fig. 1), a hepatic vascular bed, and coronary vascular beds. The generic vascular bed model is used for all microvasculature beds except the liver and myocardium (27) and is based on the commonly used three-element Windkessel model. All baseline parameters of vessel segments and vascular beds are adopted from the study by Mynard and Smolich (27).

### Time-Varying Elastance Heart Model

The relationship between the pressure and the volume of a heart chamber is given by

(*5*) $$\text{P}={\text{P}}_{\text{pc}}+\frac{E_{\text{nat}}}{E_{\text{sep}}}P^{*}+E_{\text{nat}}\left(\text{V}-{\text{ V}}_{\text{P}=0}\right)-R_{\text{s}}q,$$

where ${\text{P}}_{\text{pc}}$ is the pericardial pressure (assumed to depend exponentially on the total chamber volumes), $E_{\text{nat}}$ is the native elastance of the chamber, $E_{\text{sep}}$ is the septal elastance, ${\text{V}}_{\text{P}=0}$ is the volume of the chamber in zero pressure, $R_{\text{s}}$ is the source resistance, and $P^{\text{*}}$ is the pressure in the contralateral chamber. Parameters varied in this study and their corresponding range are listed in Table 1.

**Table 1.** Physical parameters used in this study

Physical Parameter	Baseline	Range
Aortic PWV, m/s	4.66	[4.66, 13.98]
Heart rate, beats/min	75	[30, 180]
LV end-systolic elastance, mmHg/mL	2.5	[0.6, 5.0]
LV end-diastolic volume, mL	136	[65, 465]
Ejection fraction, %	55	[14, 70]
Stroke volume, mL	74	[19, 186]
Cardiac output, L/min	5.56	[1.4, 7.1]

LV, left ventricular; PWV, pulse wave velocity.

### Hemodynamic Analysis

The total power ${\bar{P}}_{\text{total}}$ transmitted to the brain over a cardiac cycle of length *T* is calculated as the average of the product of the pressure P(*t*) and the flow *Q*(*t*). We employ pressure and flow data from the left common carotid artery for computing energy since it is the only cerebral branch directly connected to the aortic arch. The steady power ${\bar{P}}_{\text{s}}$ is computed as the product of mean pressure ${\text{P}}_{\text{mean}}$ and mean flow $Q_{\text{mean}}$ in each segment. The pulsatile transmitted power ${\bar{P}}_{\text{pulse}}$ is the difference between the total power and the steady power. Each of these power quantities is, respectively, given by

(*6*) $${\bar{P}}_{\text{total}}=\frac{1}{T}\int_{0}^{T}\text{P}\left(t\right)Q\left(t\right)\text{d}t,$$

(*7*) $${\bar{P}}_{s}={\text{P}}_{\text{mean}}Q_{\text{mean}},$$

(*8*) $${\bar{P}}_{\text{pulse}}={\bar{P}}_{\text{total}}-{\bar{P}}_{\text{s}}.$$

Total fluid flow transmitted to the cerebral network is computed by summing the average flow over one cardiac cycle (i.e., integrating over time) for all four arteries connected to the brain (two carotid and two vertebral). In addition to *Eqs. 6–8*, wave intensity (WI), a well-established clinical metric (40), is also considered to quantify energy transmission to the brain. Mathematically speaking, WI is computed as the product of the change in pressure (dP) times the change in velocity (dU) during a small interval, i.e.,

(*9*) dI=dP·dU.

To remove the dependency of dI on sampling time, the derivative of pressure and velocity are divided by the time interval (denoted as dP/dt and dU/dt, respectively), yielding units of power per unit area per unit time ($\text{W}\cdot {\text{s}}^{-2}\cdot {\text{m}}^{-2}$) (40–42). To account for changes in the diameter, we also conducted wave power analysis (43). Wave power is defined as the product of the pressure and volumetric flow signals and has the unit of the power (Watt) (44). We additionally investigated the reflection measures from wave separation analysis. To this end, the carotid pressure waveform is decomposed into its forward and backward components, following previous works (45, 46). The corresponding reflection index (RI, defined as the ratio of the peak backward pressure over the total pressure) is then computed and reported as a percentage.

As a third and final measure employed in this work, we also consider the carotid (flow) pulsatility index (CPI), a clinical parameter (2, 12) based on a single-flow waveform measurement that is defined as

(*10*) $$\text{CPI}=\frac{q_{\text{max}}-q_{\text{min}}}{\frac{1}{T}\int_{0}^{T}q\left(t\right)\text{d}t},$$

where $q_{\text{min}}\text{ and }q_{\text{max}}$ are, respectively, the minimum and maximum flow transmitted to the brain through the carotid artery during a cardiac cycle.

**Figure 2.**
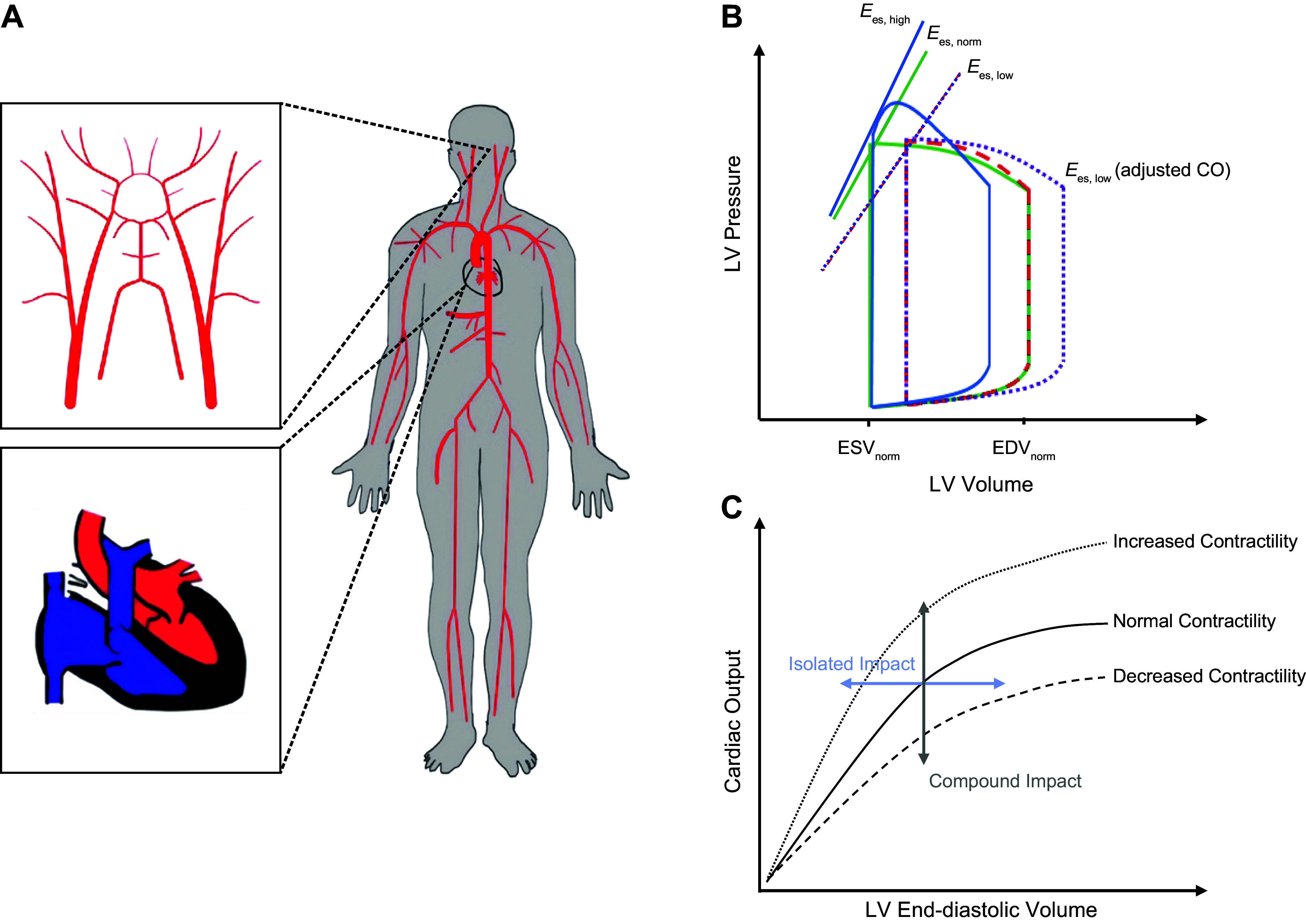
*A*: schematic representation of the human circulatory system. *B*: interventricular pressure-volume loops for different cases of contractility (demarcated in different line styles and colors). ESV and EDV, end-systolic volume and end-diastolic volume, respectively. *C*: compounded impact [a fixed left ventricular end-diastolic volume (LVEDV)] and the isolated impact [a fixed cardiac output (CO)] of contractility. *E*_es_, end-systolic elastance.

Figure 2 illustrates the impact of the elastance $E_{\text{es}}$ on the LV pressure-volume loop. For example, varying $E_{\text{es}}$ while fixing the preload and LV end-diastolic volume (LVEDV), leads to different COs. To keep the CO constant at different levels of contractility, we adjust the LVEDV (Frank–Starling mechanism).

### Statistical Analysis

We have conducted three ordinary least square regressions using a heteroscedasticity-consistent covariance matrix (HC3 type) (47) to assess the statistical significance of the relationship between the dependent variables (e.g., carotid pulsatile power) and the corresponding independent variables. Independent variables in this study are considered to be LV contractility, aortic PWV, and heart rate. All independent variables have been incorporated in the models as categorical variables. The Shapiro–Wilk test for normality has been conducted to check the normality of each regression’s residuals. The independence of the variables has also been tested by a χ^2^ test to check their correlations (no correlation has been found among the independent variables). Statistical significance is defined as α=0.05/40=0.001 (Bonferroni adjusted). The software package R version 4.2.2 has been used to conduct the statistical analysis.

## RESULTS

### Physiological Accuracy of the Model

**Figure 3.**
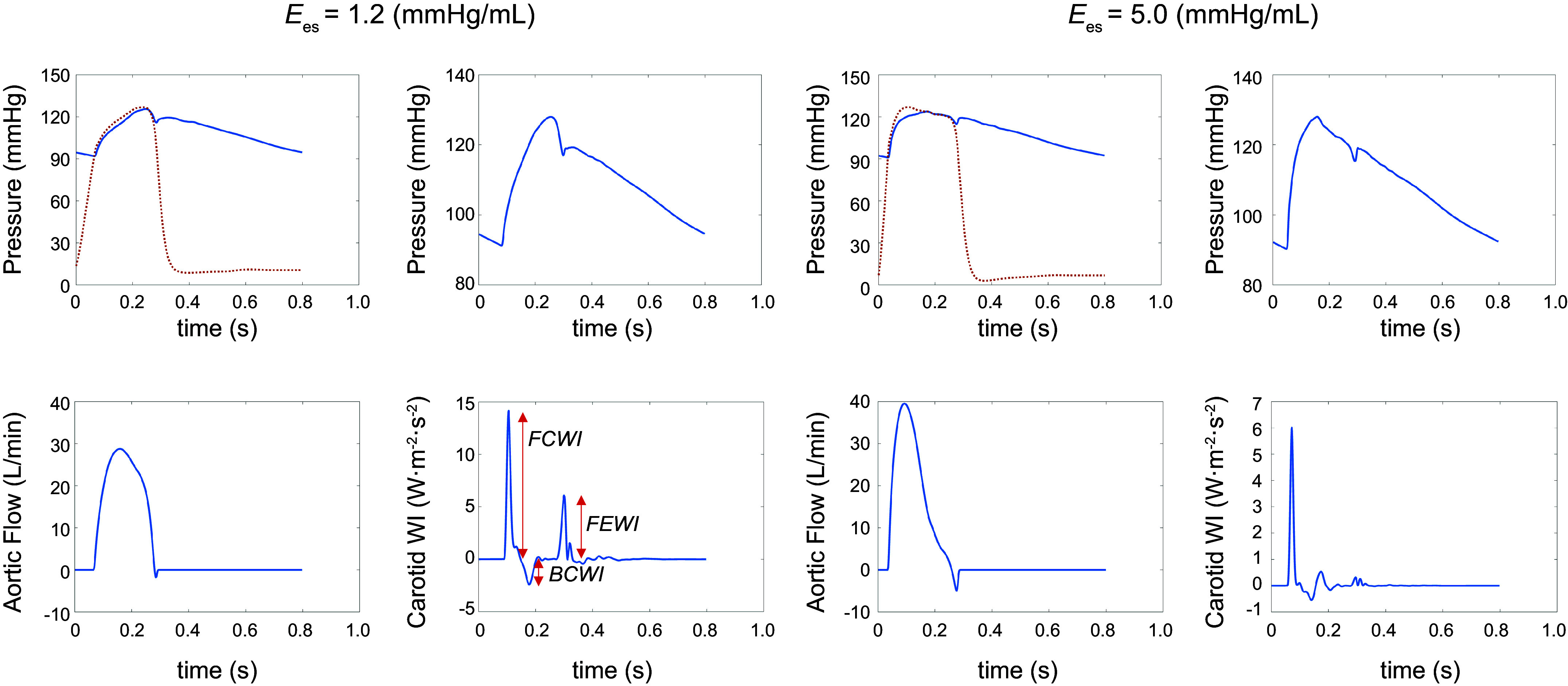
Effects of left ventricular (LV) contractility on aortic and carotid hemodynamics. Cardiac output (CO) is the same for both sets *right* and *left*. *Left*: impact of reduced contractility. *Right*: impact of increased contractility. BCWI, backward compression wave intensity; *E*_es_, end-systolic elastance; FCWI, forward compression wave intensity; FEWI, forward expansion wave intensity; WI, wave intensity.

Figure 3 presents various pressure and flow waveforms, simulated via the numerical methodology described earlier, for cases of decreased (Ees=1.2 mmHg/mL) and increased (Ees=5.0 mmHg/mL) contractility, where LVEDV is adjusted to have the same CO for both (5.60 L/min). These cases are computed at a baseline heart rate (75 beats/min) and aortic PWV (4.66 m/s), both within physiological ranges. The presented pressure and flow waveforms demonstrate the expected dynamics of the LV and the aorta during systole, including the presence of the pressure dicrotic notch as well as the physiological point-to-point consistency of the pressure with the flow. For the case of increased contractility, all waveforms have steeper upstrokes at the onset of ejection and reach their respective peaks earlier in systole. Even though varying LVEDV preserves CO, the peak flow is significantly higher for the increased contractility case. In addition, while pulse pressure minimally changes, the corresponding shape of the pressure waveform is affected by changes in contractility alone.

Figure 3 further presents the computed carotid WI for the decreased and increased contractility cases. The curves fully capture the typical pattern of WI (40, 41, 48): a large-amplitude forward (positive) peak corresponding to the initial compression caused by LV contraction (forward compression wave intensity, or FCWI); a subsequent small-amplitude backward (negative) peak corresponding to the reflection of the initial contraction (backward compression wave intensity, BCWI); and a final moderate-amplitude forward decompression wave in protodiastole (forward expansion wave intensity, FEWI). The overall results of Fig. 3 demonstrate the general ability of our in silico computational model to reproduce the physiological characteristics of the LV, the aorta, and the carotid artery.

### Effect of LV Contractility on Transmitted Pulsatility to the Brain

**Figure 4.**
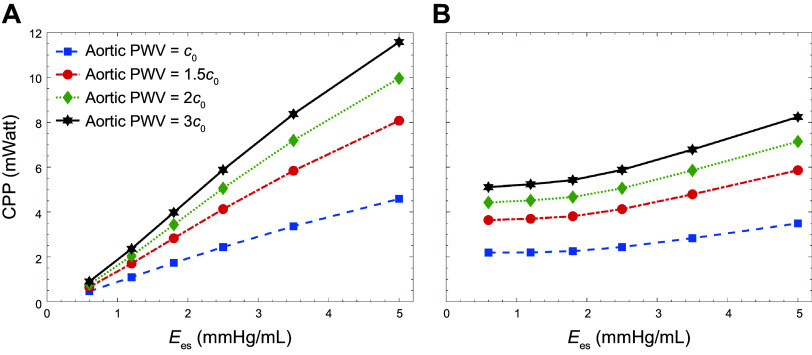
Carotid pulsatile power (CPP) per cardiac cycle vs. contractility [as measured by end-systolic elastance (*E*_es_)] at different levels of aortic stiffness at fixed left ventricular end-diastolic volume [LVEDV, changing cardiac output (CO); *A*] and fixed CO (*B*).

Figure 4*A* presents the carotid pulsatile power (CPP) transmitted to the brain as a function of contractility for different levels of aortic PWV at a fixed LVEDV. The data are computed at the baseline heart rate (75 beats/min). Since LVEDV is fixed, changes in contractility lead to corresponding changes in CO (see also Fig. 2), further compounding the overall effect of varying contractility by $E_{\text{es}}$. Figure 4*B* presents the isolated impact of contractility at a fixed CO (achieved by adjusting LVEDV) on the transmitted pulsatile power to the brain, where it can be observed that, at all values of $E_{\text{es}}$, pulsatile power in the carotid artery increases as a function of aortic PWV.

**Figure 5.**
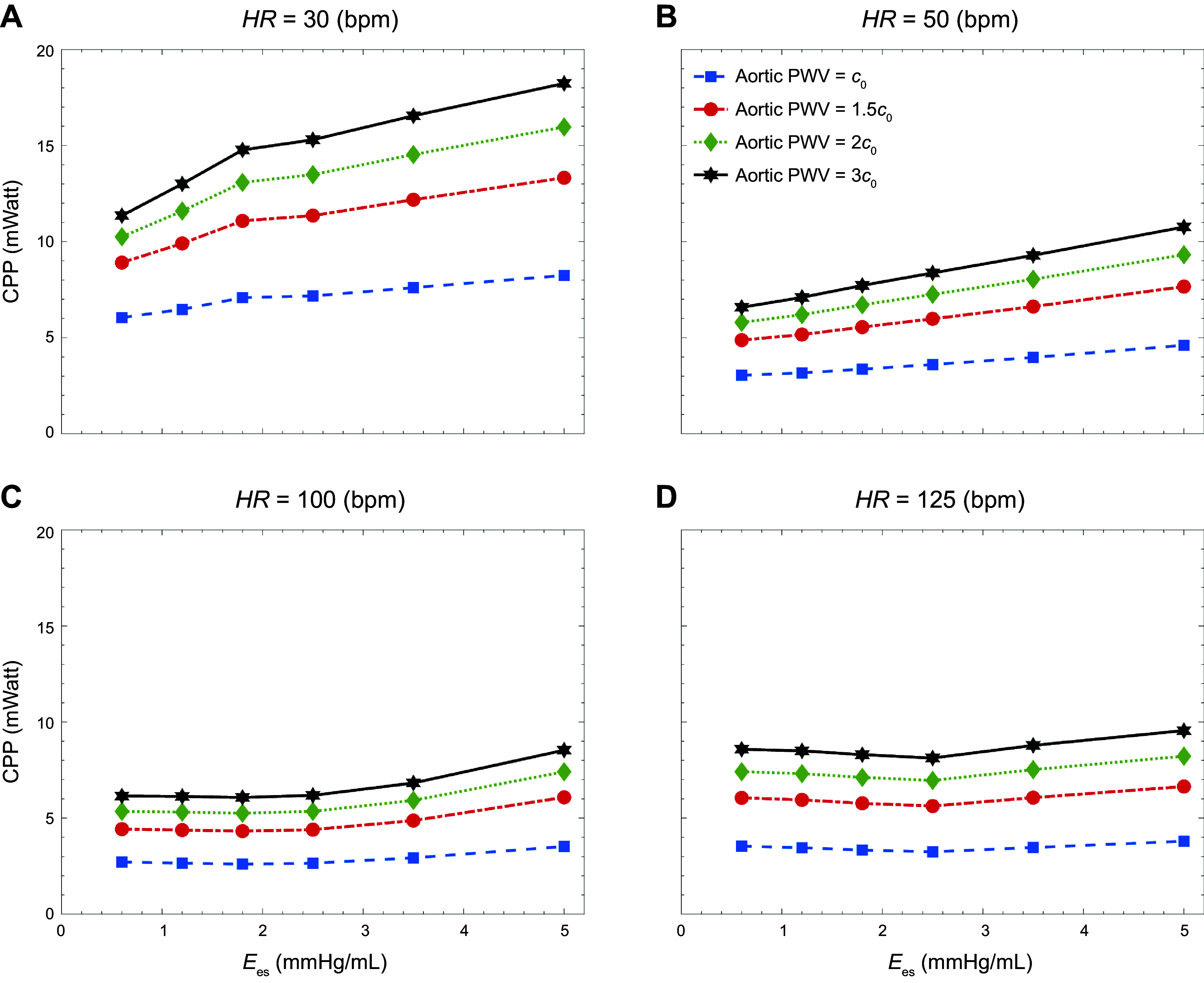
Carotid pulsatile power (CPP) per cardiac cycle vs. contractility [as measured by end-systolic elastance (*E*_es_)] at different levels of aortic stiffness at other heart rates (HR; in beats/min): 30 (*A*), 50 (*B*), 100 (*C*), and 125 (*D*).

Figure 5 presents CPP as a function of contractility ($E_{\text{es}}$) at different levels of both aortic PWV and heart rate (HR). As before, to achieve a constant CO (5.6 L/min) at baseline aortic PWV (${\text{c}}_{0}$), values of LVEDV are accordingly adjusted; hence, at each PWV, the changes in CPP are a consequence of the isolated changes in contractility. Results demonstrate a trend toward increased transmitted pulsatile power to the brain as contractility increases. However, the rate of this increase depends on HR. Table 2 additionally presents a comparison between baseline and increased aortic PWV (which can result from aging) on CPP transmitted to the brain and further presents corresponding values of the aortic pulsatility index (CPI) computed using *Eq. 10*.

**Table 2.** Impact of LV contractility at two levels of aortic stiffness on the transmitted pulsatility to the brain

Contractility, mmHg/mL	0.6	1.2	1.8	2.5	3.5	5.0
Baseline aortic PWV, ${\text{c}}_{0}$						
Carotid pulsatile power, mW	2.19	2.20	2.25	2.44	2.83	3.49
Carotid pulsatility index	5.00	4.96	4.96	5.04	5.90	7.55
Increased aortic PWV, $3{\text{c}}_{0}$						
Carotid pulsatile power, mW	5.11	5.24	5.42	5.88	6.78	8.24
Carotid pulsatility index	8.50	8.52	8.61	8.86	9.43	11.16

All values are reported at a heart rate of 75 beats/min. LV, left ventricular; PWV, pulse wave velocity.

### Effect of Heart Rate on Transmitted Pulsatility to the Brain

**Figure 6.**
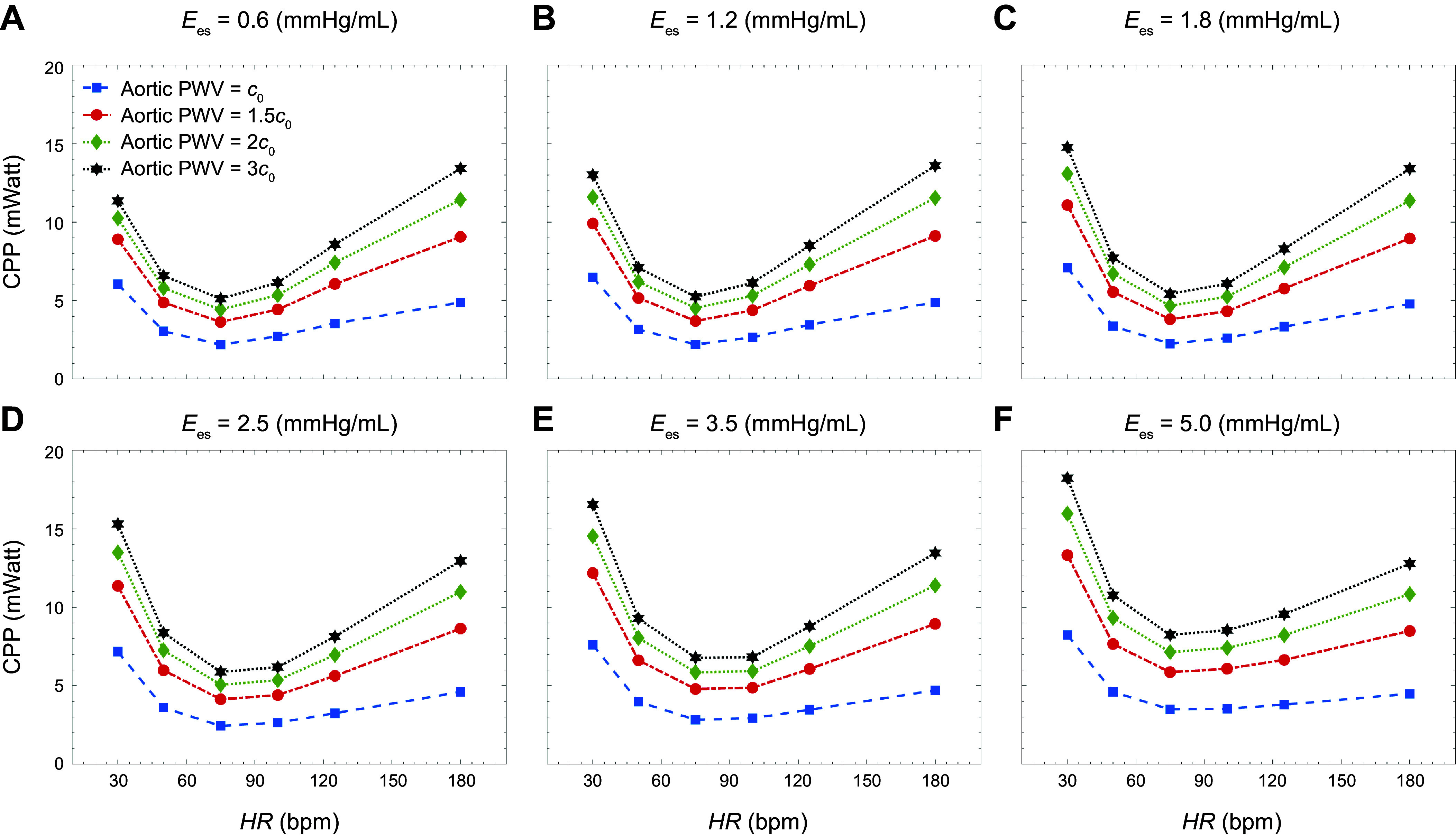
Carotid pulsatile power (CPP) per cardiac cycle vs. heart rate (HR) at different levels of aortic stiffness: end-systolic elastance (*E*_es;_ in mmHg/mL), 0.6 (*A*), 1.2 (*B*), 1.8 (*C*), 2.5 (*D*), 3.5 (*E*), and 5.0 (*F*).

Figure 6 presents values of CPP as a function of HR for different levels of aortic PWV. The data in each plot are obtained at different levels of contractility (as measured by $E_{\text{es}}$). CO is fixed at each level of aortic PWV in a manner as has been described before. As HR increases, CPP decreases until the heart rate reaches an optimum point corresponding to where CPP is minimized. CPP increases with HR beyond this optimum point. Note that this phenomenon is present for all the different multiplicative factors of aortic PWV considered here, as well as for all the different levels of contractility. In all cases, the optimum point is located near the normal human heart rate (75 beats/min). The *P* values from a corresponding Shapiro–Wilk test are too large to reject the null hypothesis of the normality of the residuals. The coefficients of the regression results yield the expected signs and magnitudes. In addition, by experimental design, our independent variables are uncorrelated; however, we have also conducted a χ^2^ test to check their correlations, and the results confirm the hypothesis (see appendix for details). Indeed, the outcome of the statistical analysis reveals that an increase from the initial level of contractility ($E_{\text{es}}$ = 0.6 mmHg/mL) to a contractility level of $E_{\text{es}}$ = 1.2 mmHg/mL or $E_{\text{es}}$ = 1.8 mmHg/mL is not statistically significant for CPP (see appendix). On the other hand, the rest of the coefficients for other variables are significant, and all signs are found to be as expected. The impact of a change in HR is significant at all levels. Furthermore, CPP rises as the level of PWV rises, and all results are statistically significant (see appendix).

**Figure 7.**
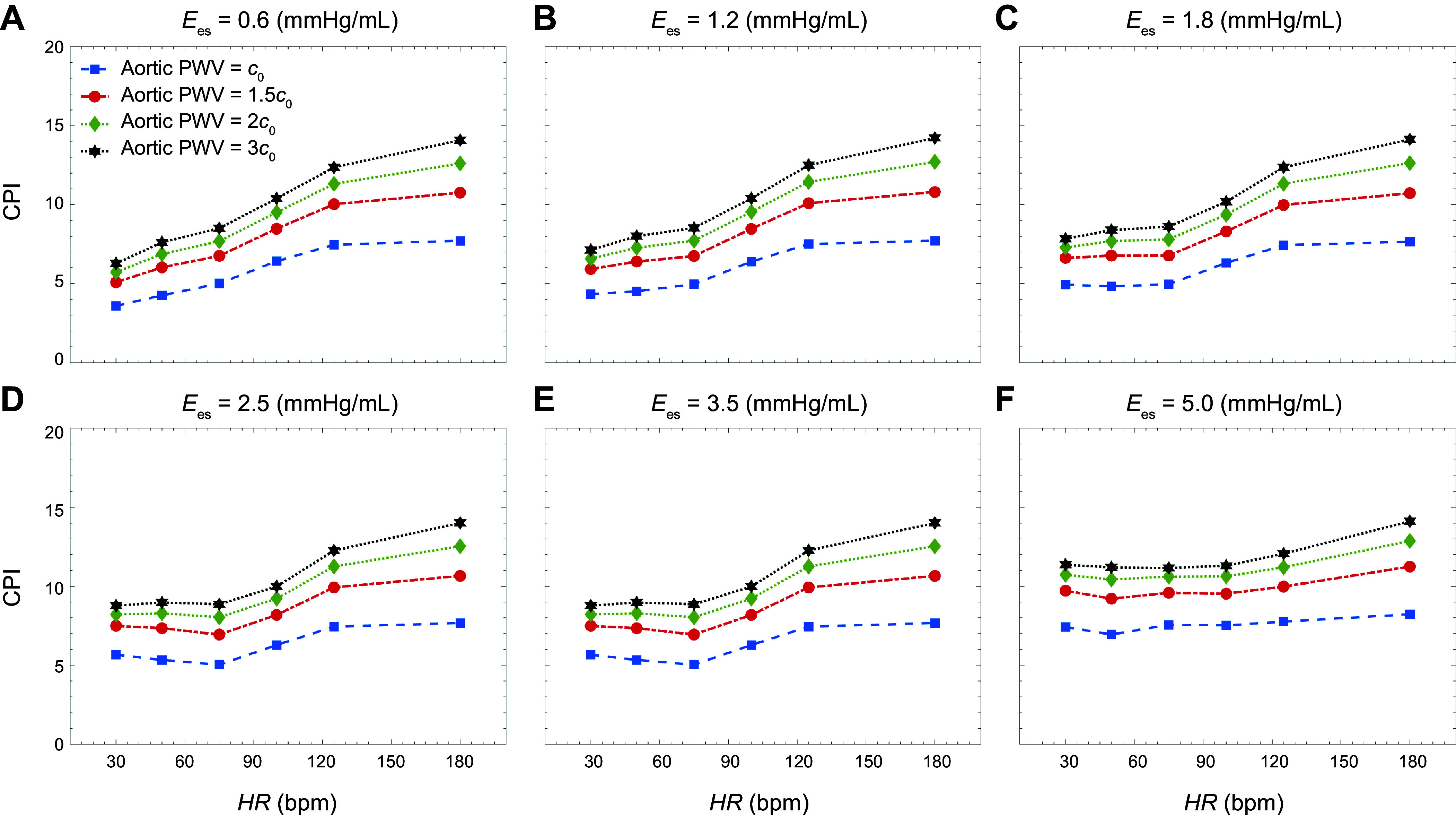
Carotid pulsatility index (CPI) per cardiac cycle vs. heart rate (HR) at different levels of aortic stiffness: end-systolic elastance (*E*_es;_ in mmHg/mL), 0.6 (*A*), 1.2 (*B*), 1.8 (*C*), 2.5 (*D*), 3.5 (*E*), and 5.0 (*F*).

Figure 7 presents the carotid pulsatility index (CPI) as a function of HR for different levels of aortic PWV. The data in each plot are obtained at different levels of contractility (as measured by $E_{\text{es}}$). As before, CO is fixed at each level of aortic PWV. The results suggest a trend toward increased CPI as HR increases. The statistical results (see appendix) for CPI demonstrate that CPI increases when independent variables increase; however, the impact at the lower level of HR and contractility does not show statistical significance.

### Effect of LV-Aorta Dynamics on Wave Intensity

**Figure 8.**
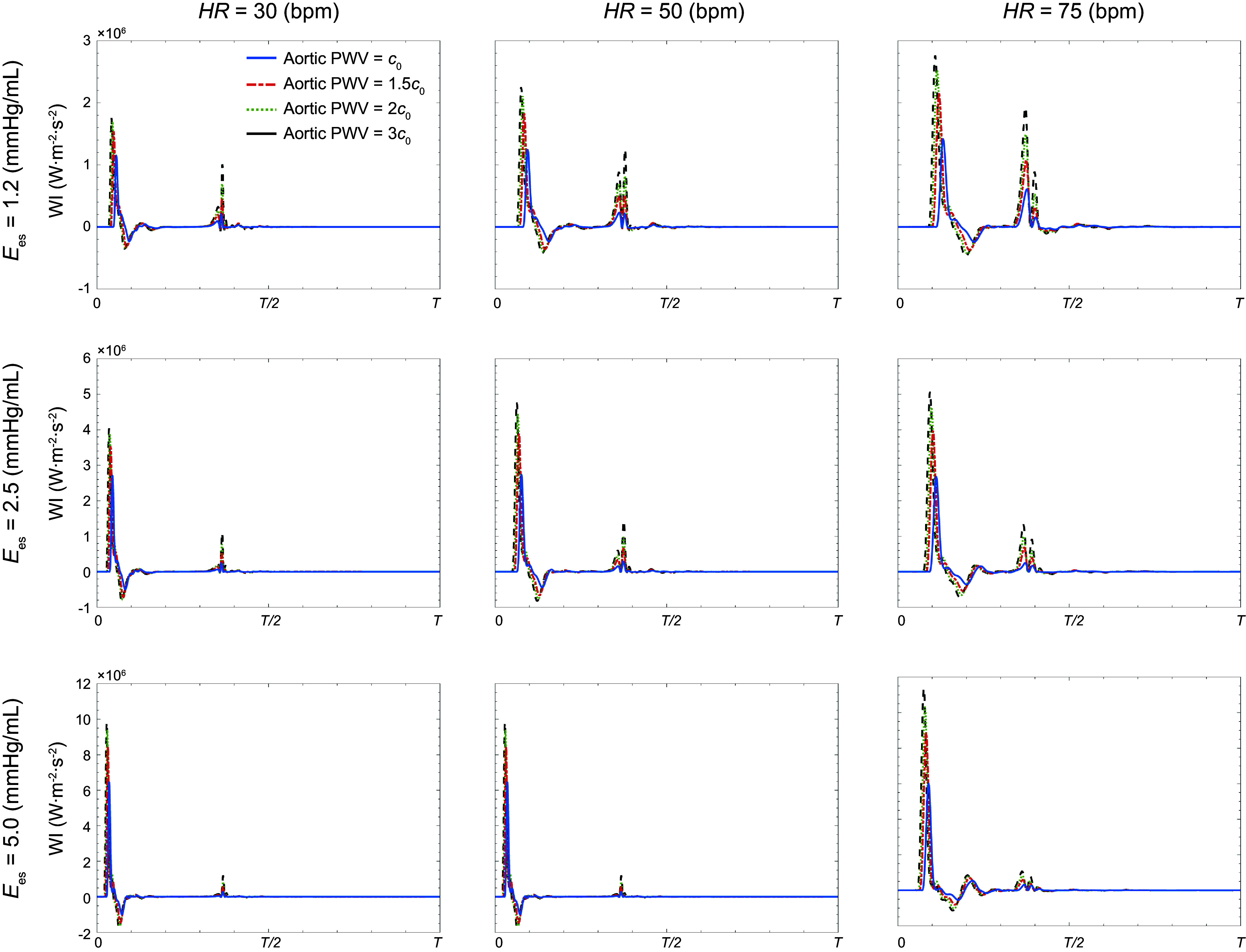
Sample carotid wave intensity (WI) patterns at different heart rate (HR) and different levels of contractility [as measured by end-systolic elastance (*E*_es_)]. Each plot contains data obtained at different levels of aortic stiffness [quantified by pulse wave velocity (PWV)].

**Figure A3.**
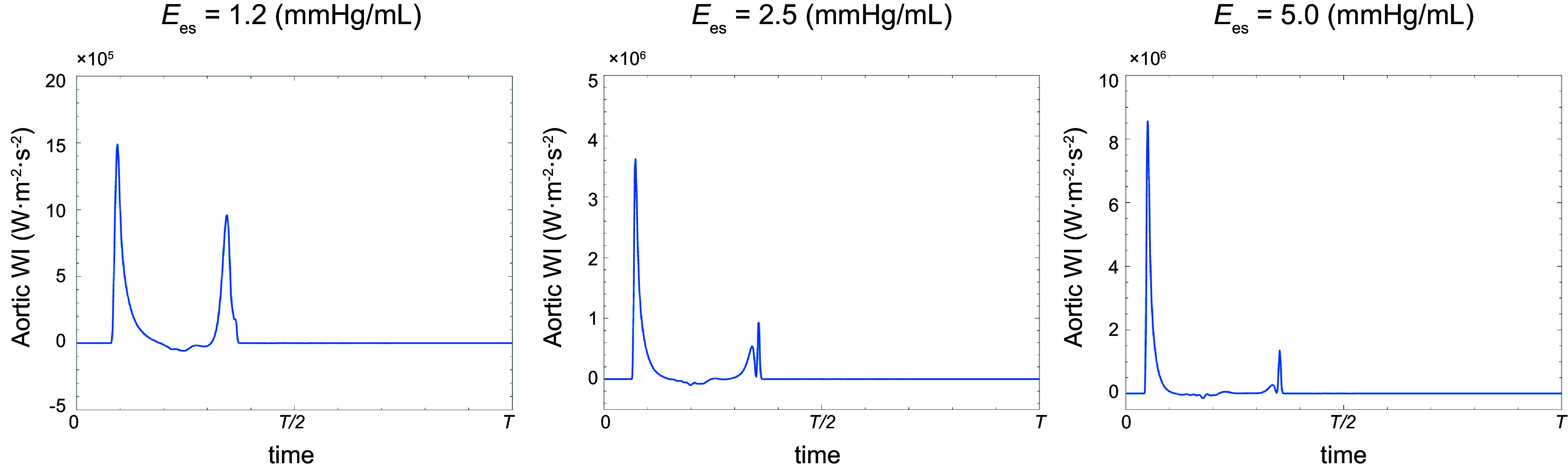
Sample aortic wave intensity (WI) patterns at different levels of contractility [as measured by end-systolic elastance (*E*_es_)].

Figure 8 presents calculated carotid WI patterns from simulations at different levels of aortic PWV for different HR and contractility values. Similarly to Fig. 3, these patterns capture all the well-known fiducial features (40), including the large-amplitude forward (positive) peak FCWI that is followed in sequence by both a small-amplitude backward (negative) peak BCWI and a moderate-amplitude forward decompression wave FEWI. Sample patterns of aortic wave intensity at different levels of contractility are presented in appendix (Fig. A3).

Table 3 presents peak amplitudes of the major features of WI (FCWI, BCWI, and FEWI) at different levels of contractility. The data are presented at normal HR for both baseline aortic PWV and an increased PWV. For reference, we have also included the amplitude of the first peak of the wave power (forward compression wave power; FCWP) in Table 3. The reflection index (as a percentage) at different contractility values, determined from carotid pressure wave separation, is also reported in Table 3.

**Table 3.** Impact of LV contractility on carotid WI and WP indices, as well as the reflection index at different aortic stiffness (PWV)

Contractility, mmHg/mL	0.6	1.2	1.8	2.5	3.5	5.0
Baseline PWV ${\text{c}}_{0}$						
FCWI, W·m^−2^·s^−2^ × 10^5^	9.5	14.2	19.4	26.7	39.0	60.2
BCWI, W·m^−2^·s^−2^ × 10^5^	1.7	2.4	2.9	3.6	4.4	5.2
FEWI, W·m^−2^·s^−2^ × 10^5^	8.8	6.1	3.8	2.5	2.9	5.5
FCWP, W × 10^−6^	39.5	59.7	82.4	111.5	170.9	262.7
Reflection index, %	35.5	35.2	35.4	35.5	36.9	38.6
Increased PWV $3{\text{c}}_{0}$						
FCWI, W·m^−2^·s^−2^ × 10^5^	18.7	27.6	37.1	50.7	74.0	113.0
BCWI, W·m^−2^·s^−2^ × 10^5^	3.4	4.6	5.8	7.1	8.9	11.4
FEWI, W·m^−2^·s^−2^ × 10^5^	8.9	19.0	15.6	13.1	11.6	10.6
FCWP, W × 10^−6^	75.9	112.4	153.2	211.7	314.9	498.0
Reflection index, %	38.2	38.6	39.2	39.4	39.6	40.5

All values are reported at a heart rate of 75 beats/min. The effect of contractility is isolated at fixed cardiac output. BCWI, backward compression wave intensity; FCWI, forward compression wave intensity; FCWP, forward compression wave power; FEWI, forward expansion wave intensity; LV, left ventricular; PWV, pulse wave velocity; WI, wave intensity; WP, wave power.

Table 4 presents peak amplitudes of the major features of WI (FCWI, BCWI, and FEWI) at different HR. The data are presented for both baseline aortic PWV and an increased PWV at a state of normal contractility ($E_{\text{es}}$ = 2.5 mmHg/mL). Similarly, to Table 3, we have also included the amplitude of the first peak of the wave power (FCWP) in Table 4. The reflection index for the carotid pressure at different values of heart rate is also reported Table 4.

**Table 4.** Impact of heart rate on carotid WI and WP indices, as well as the reflection index at different aortic stiffness (PWV)

Heart rate, beats/min	30	50	75	100	125
Baseline PWV ${\text{c}}_{0}$					
FCWI, W·m^−2^·s^−2^ × 10^5^	27.1	27.3	26.7	28.3	28.9
BCWI, W·m^−2^·s^−2^ × 10^5^	4.9	4.4	3.5	3.1	2.5
FEWI, W·m^−2^·s^−2^ × 10^5^	2.5	2.9	2.5	6.8	19.9
FCWP, W × 10^−6^	106.3	112.2	114.9	123.3	127.7
Reflection index, %	42.6	39.3	35.5	36.2	36.6
Increased PWV $3{\text{c}}_{0}$					
FCWI, W·m^−2^·s^−2^ × 10^5^	40.4	47.7	50.7	55.2	58.2
BCWI, W·m^−2^·s^−2^ × 10^5^	8.1	8.2	7.1	6.4	5.5
FEWI, W·m^−2^·s^−2^ × 10^5^	10.9	14.3	13.2	18.6	46.3
FCWP, W × 10^−6^	151.0	190.9	211.7	235.2	252.4
Reflection index, %	43.7	42.2	39.4	39.2	39.4

All values are reported at a contractility of 2.5 mmHg/mL. BCWI, backward compression wave intensity; FCWI, forward compression wave intensity; FCWP, forward compression wave power; FEWI, forward expansion wave intensity; PWV, pulse wave velocity; WI, wave intensity; WP, wave power.

### Effect of LV-Aorta Dynamics on Brain Perfusion

**Figure 9.**
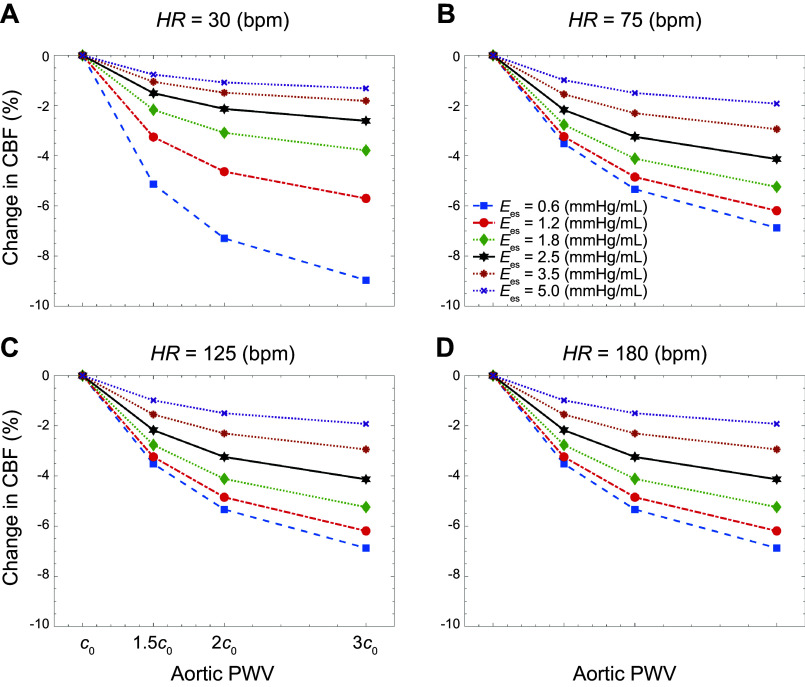
Change in the transmitted flow to the brain vs. aortic stiffness [as measured by pulse wave velocity (PWV)] at different levels of contractility: heart rate (HR; in beats/min), 30 (*A*), 75 (*B*), 125 (*C*), and 180 (*D*). CBF, cerebral blood flow; *E*_es_, end-systolic elastance.

Figure 9 demonstrates how changes in aortic stiffness (as measured by PWV) at different levels of contractility affect transmitted cerebral blood flow (CBF). The data in each plot are obtained at different heart rates. At each wave state, the percent change is computed by the change in flow relative to baseline PWV. Note that at each contractility, LVEDV remains fixed. Results suggest a trend toward decreased cerebral flow as aortic stiffness increases. The rate of this change depends on the contractility and HR. The regression results (see appendix) indicate that CBF decreases monotonically as the level of PWV increases, where the coefficients are statistically significant.

## DISCUSSION

In this study, we have investigated the effect of LV-aortic dynamics on brain perfusion and the transmission of excessive wave pulsatility to cerebral circulation. We have modeled age-related changes in the arterial system and cardiac dynamics. Our results suggest that *1*) LV contractility by itself affects the pulsatile energy transmission to the brain (even at a preserved cardiac output); *2*) at different levels of LV contractility and aortic stiffness, there exists an optimum wave condition, occurring near the normal human heart rate (75 beats/min), in which excessive pulsatile energy (power) transmission to the brain is minimized; and *3*) at a given heart rate and LV contractility, greater aortic stiffness leads to lower cerebral blood flow. At the limit of brain autoregulation, the compensatory mechanism for adjusting the cerebral flow is achieved either by increasing the LV contractility or the heart rate. Our results suggest that the former consistently leads to higher pulsatile power transmission. The latter can either increase (for values less than normal heart rate) or decrease (for values beyond normal heart rate) pulsatile power transmission to the brain.

### Impact of LV Contractility

We have used a reduced-order 1-D model of the entire human circulation to study and elucidate the underlying mechanisms involved in LV-aorta-brain hemodynamic coupling. The numerical solver employed in this work incorporates the nonlinear and nonstationary coupling of various cardiovascular system components [including an ordinary differential equation (ODE)-based 4-chamber heart model with valves], where the validation results that have been presented support its suitability for the objectives of this study. Results further demonstrate that our model can adequately capture the effects of contractility on central and peripheral pressure waveforms (Fig. 3). The expectedly steeper upstrokes in both pressure and aortic flow waveforms for increased contractility are well captured in this model and are consistent with previous studies (22). Our findings suggest that increased LV contractility alone can directly alter central and peripheral hemodynamics, even for unchanged arterial loads and cardiac outputs. These observations are consistent with previous experimental and clinical studies (13, 49).

The first principal finding in this study is related to examining the impact of LV contractility on transmitted energy and pulsatility to the cerebral network, where we have used end-systolic elastance ($E_{\text{es}}$) as a measure to quantify the state of LV contractility. Figure 4 demonstrates the true effect of contractility on pulsatile energy transmission to the brain (where CO is fixed by adjusting LVEDV; see Fig. 2), where our results suggest that even at a fixed CO, an increase in contractility alone can lead to elevated levels of harmful pulsatile energy transmission to the brain. This behavior is also a function of aortic stiffness (Fig. 4). However, the rate of increase in pulsatile energy transmission as a function of contractility is slower when the CO is compensated for than when the CO is affected by changes in $E_{\text{es}}$. In other words, the impact of contractility on the cerebral pulsatile power depends on the preload (measured by LVEDV) as well. As preload is adjusted to compensate for CO, the effect of contractility is less pronounced. Since CO and the total arterial resistance of the system is the same for different levels of contractility (at the same aortic PWV), the steady portion of the transmitted power does not change (13). However, since the shape of the pressure also changes because of contractility, the total power increases (*Eq. 6*), leading to an increase in the transmitted pulsatile power (*Eq. 8*).

Results also suggest that the impact of contractility on brain perfusion depends on heart rate (Fig. 5 and Table 2). At lower heart rates, changes in carotid pulsatile power are more pronounced than at higher heart rates. At a fixed travel time (keeping PWV constant), changing the heart rate affects the interaction between the compression waves generated by the LV and the reflected waves due to vessel branching (30, 50). The sample net effect of these two types of waves is illustrated in the WI patterns of Figs. 3 and 8. These interactions become less sensitive to contractility at higher heart rates. Hence, the pulsatile portion of the power varies less. This pattern can be observed at all levels of aortic stiffness considered in this work. Another major finding in this study is the relation between volume blood flow transmission to the brain and heart contractility, as shown in Fig. 9. Results suggest that decreased contractility at fixed heart rate and aortic PWV leads to a reduction in volume blood flow transmission (brain malperfusion). This can explain one of the underlying mechanisms involved in heart failure-induced brain injury (where LV contractility is impaired).

### Presence of an Optimum Heart Rate

Our results indicate that there is an optimum heart rate at which the transmitted carotid pulsatile energy is minimized (Fig. 6). This is consistent with previous findings (21). Pulsatile energy decreases with increasing heart rate until it reaches this minimum value. Beyond this value, waves transmitted to the brain begin to act destructively, and, as a result, pulsatile power starts increasing as the heart rate increases. This has implications for the aging population, where resting HR generally increases (51, 52). In addition, aging leads to the stiffening of the aorta, further increasing the pulsatile energy transmission to the brain. Indeed, our findings are consistent with previous studies (2, 50) suggesting that aortic wave optimization is one of the key design characteristics in the mammalian cardiovascular system. To the best of our knowledge, the presence of the optimum heart rate at different levels of contractility in the LV-aorta-brain system has not been reported in prior studies (including from in vivo experiments).

In contrast to pulsatile energy, which requires both flow and pressure waveforms to calculate, the carotid flow pulsatility index (CPI) is a conventional dimensionless parameter quantifying hemodynamic pulsatility transmission to cerebrovasculature based only on flow measurements (2, 12). Figure 7 presents CPI values corresponding to the same values of heart rate, contractility, and aortic PWV considered for our pulsatile energy analysis. A trend of increasing CPI with increasing heart rate can be observed in all cases. However, the CPI curves do not capture the nonlinearity and the presence of a minimum that can be found in the CPP curves of Fig. 6. A statistical analysis of the coefficients reveals that the impact of lower levels of heart rate is statistically significant on CPI, although this impact does not show statistical significance on the CPI results (Tables A2 and A3 in appendix). This suggests that considering the flow waveform alone may not be adequate in properly quantifying the pulsatility transmitted to the brain and that consideration of energy-based methods that include both pressure and flow; hence, we suggest that future clinical studies should include both indices in their assessments. This is particularly prudent since aortic aging simultaneously affects the transmitted flow and pressure waves to the brain (21).

### Impact of Aortic Stiffness (Vascular Aging)

Aortic stiffening is the primary cause of systolic hypertension with aging (53). It has been shown that blood pressure lowering with antihypertensive agents compared with control is significantly associated with a lower risk of incident dementia or cognitive impairment (54). In prior work, we have shown such age-related stiffening (11, 21) is a powerful predictor of insult to the microvasculature in the brain, more so than blood pressure (15). Our results in this work confirm that aortic stiffening does indeed increase the transmission of harmful pulsatility to the brain. This excessive pulsatility can be observed at all contractility states in both CPP (Fig. 6) and CPI (Fig. 7). For example, age-related changes in aortic biomechanics can lead to an increase from the average aortic PWV of 7 m/s for a young adult to an average aortic PWV of 14 m/s for an old individual (55). This age-related increase in aortic PWV under normal contractility leads to a 42% increase in pulsatile energy transmission to the brain. The effect of these age-related changes becomes even more pronounced when compounded with increased contractility. For example, changes in aortic PWV and contractility from normal values for a young adult to increased values for an old individual can elevate the pulsatile energy transmission to the brain more than 200%.

Results from Fig. 9 further suggest that greater aortic stiffness leads to lower cerebral blood flow at a fixed heart condition (i.e., a fixed contractility). A statistical analysis confirms a significant dependency for cerebral blood flow on aortic stiffness (Table A4 in appendix). These findings are consistent with a recent population-based clinical study by Jefferson et al. (56), where it was reported that more significant aortic stiffening relates to lower cerebral blood flow, especially among individuals with an increased genetic predisposition for Alzheimer’s disease. The authors have hypothesized that this mechanism is due to microcirculatory remodeling in response to higher pulsatility in the cerebrovascular network. In our present investigation, an isolated increase in aortic stiffness (under fixed contractility) leads to decreased blood flow transmission to the brain (56). Therefore, the effects of harmful carotid pulsatile energy transmission are likely compounded with the decreased flow transmission to the brain as a result of such age-related aortic stiffening.

**Figure 10.**
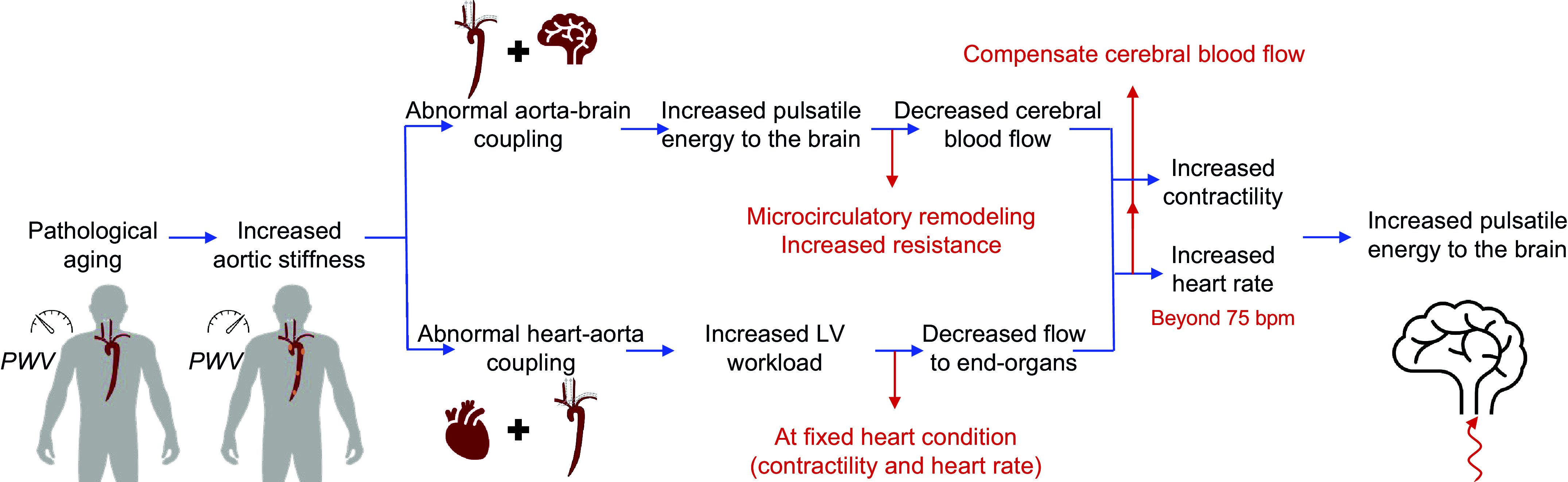
Mechanistic model, based on the findings of this study, for compensating cerebral blood flow due to pathological aging. LV, left ventricular; PWV, pulse wave velocity.

A decreased cerebral blood flow, which can result from systemic diseases such as heart failure, can contribute to the dysfunction of the neurovascular unit (17). This is the prevailing view of the underlying mechanism of HF-induced Alzheimer’s disease (16). Our results suggest that at low levels of LV contractility, the reduction in cerebral blood flow due to age-related changes in aortic stiffness becomes even more pronounced (Fig. 9). To counteract this suboptimal flow, the brain may employ its autoregulatory mechanism by reducing the resistance. However, this effort to maintain perfusion may have other deleterious effects, such as allowing further penetration of pulsatile energy into the microvasculature where it may cause more damage (8). In addition, the autoregulatory capacity of the brain may be limited in the setting of microvascular disease (1, 8, 10). Under these circumstances, the body needs to either increase the heart rate or the contractility to compensate for and to regulate the blood flow to the brain. The latter leads to higher harmful pulsatile energy transmission to the brain (Figs. 4 and 5). On the other hand, increasing the heart rate can both decrease or increase this energy transmission (Fig. 6), depending on whether the increasing HR is approaching or diverging from the minimum, respectively. Since the resting heart rate in the elderly is usually higher than the normal human heart rate (i.e., the latter case), an increase in pulsatile energy transmission will be observed (which, as explained before, can have detrimental effects on brain structure). Overall, our results demonstrate that age-related aortic stiffening can lead to a cascade of detrimental effects, because of changes in contractility and HR, on both cerebral perfusion and pulsatile energy transmission to the brain. Figure 10 summarizes this compensatory mechanism for cerebral blood flow in the context of heart-aorta-brain coupling based on the findings of this study.

### Pulse Wave Analysis

Wave intensity (WI) analysis is a well-established method for quantifying the energy carried in arterial waves (48, 57–59). In a recent population-based clinical study, Chiesa et al. (60) showed that elevated carotid WI, captured in FCWI amplitudes (e.g., in Fig. 3), predicts faster cognitive decline in long-term follow-ups independently of other cardiovascular risk factors. Their findings suggest that exposure to increased WI in mid- to late-life may contribute to the observed association between arterial stiffness in midlife and the risk of dementia in the following decades. This cannot be detected using common carotid phenotypes (60). Our results are consistent with such observations, where one can observe that elevated aortic stiffness leads to higher WI (Fig. 8 and Table 4). Our results also demonstrate that elevated FCWI not only depends on aortic stiffness but also strongly upon LV contractility (Fig. 8 and Table 3). This can be mainly attributed to the larger dP/dt resulting from increased contractility, which manifests as sharper pressure slopes in early systole and more pronounced forward compression waves (Tables 3 and 4). Similar trends can also be observed in FCWP amplitudes. The difference between wave intensity and wave power is rooted in their definition; the latter is not sensitive to variations in cross-sectional area, and thus, it is conserved at junctions (43, 44). Our results suggest that these patients might also suffer from excessive FCWI and FCWP, which could have adverse effects on brain structure (1–3, 15, 60). Tables 3 and 4 also present results for the computed reflection index at different contractility values and at different levels of the heart rate. Our results indicate that elevated aortic stiffness leads to an increase in the reflection index (Tables 3 and 4). Results also suggest that reflection index is higher at lower heart rates (below 75 beats/min) and increased contractility (above 2.5 mmHg/mL). Both these conditions may have adverse effects on cerebral circulation, particularly in terms of pulsatile energy transmission to the brain (Figs. 5 and 6).

### Limitations

The primary limitation of this study is in the vessel wall assumptions of the 1-D vasculature model formulation, i.e., neglecting the viscoelasticity [which may be important to consider in certain vessels (58, 61)]. However, our model still employs an effective nonlinear/hyperelastic wall model that has been shown previously to be appropriate under normal physiological conditions and does not lead to considerable differences with viscoelastic considerations (34). In addition, we have not included any autoregulatory models for brain circulation. However, since this study aims to investigate the impact of LV-aorta dynamics on general brain hemodynamics, the feedback response of the brain on such dynamics is beyond the scope of this work. Finally, while we examined a large range of the heart rates in our study (from 30 beats/min to 180 beats/min), the mechanistic model that we propose here is based on a stable heart rate. Future work can focus on the impact of irregular heart rate that can happen under certain cardiovascular conditions such as atrial fibrillation.

### Conclusion

We have demonstrated that alterations in LV contractility can affect pulsatile energy transmission to the brain. We have additionally demonstrated an optimum wave condition, existing at different levels of contractility, heart rate, and aortic stiffness, that minimizes this harmful pulsatile energy. We have shown that this optimum condition occurs near the normal human heart rate and remains constant across a wide range of aortic arch stiffnesses and LV contractility. Our findings also suggest that greater aortic stiffness leads to higher pulsatile energy transmission to the brain and to lower cerebral volume blood flow. These principal findings not only demonstrate the level of coupling in the LV–aorta–brain system but also demonstrate how such coupling is affected by age-related changes to the cardiovascular system. Understanding the underlying physical mechanisms involved is an important step toward developing improved therapeutic strategies for vascular-related neurodegenerative diseases. All in all, the insight from our work is central to answering questions about proper management of blood pressure and aortic stiffness among the elderly. Our results suggest that the age-related changes in aortic stiffness and contractility both affect the cerebral blood flow. Hence, it is crucial to consider the impact of antihypertensive treatment on these age-related factors and the resulting impact on cerebral blood flow. Ultimately, our findings suggest that more rational and individualized antihypertensive therapy (e.g., not only based on systolic pressure level) is needed to preclude such cerebrovascular events.

## DATA AVAILABILITY

Data will be made available upon reasonable request.

## GRANTS

A. Aghilinejad is the recipient of an American Heart Association Predoctoral fellowship. N. Pahlevan acknowledges support from the National Science Foundation CAREER Grant 2145890. This study was partially funded by the National Institute on Aging Grant 1R56AG068630-01.

## DISCLOSURES

No conflicts of interest, financial or otherwise, are declared by the authors.

## AUTHOR CONTRIBUTIONS

A.A., K.S.K., and N.M.P. conceived and designed research; A.A. performed experiments; A.A., F.A., and S.P.M. analyzed data; A.A., K.S.K., and N.M.P. interpreted results of experiments; A.A. prepared figures; A.A. drafted manuscript; A.A., F.A., S.P.M., K.S.K., and N.M.P. edited and revised manuscript; A.A., F.A., S.P.M., K.S.K., and N.M.P. approved final version of manuscript.

## Figures and Tables

**Figure A1.**
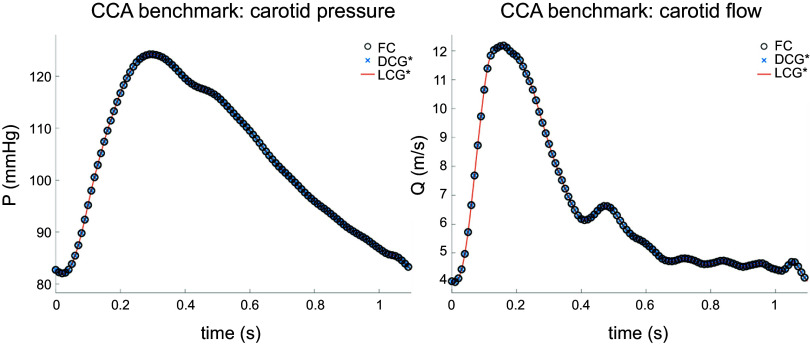
Single segment benchmark. Pressure (*left*) and flow rate (*right*) at the midpoint of the common carotid artery (CCA) simulated by Fourier continuation (FC; the solver used in this work), discontinuous Galerkin (DCG) (37), and locally conservative Galerkin (LCG) (64) methods are shown. Lines are plotted at a resolution of Δ*t* = 10^−3^ s, and markers are plotted every 10 such time steps (10^−2^ s) for easier visualization. *Provided in Boileau et al. (37).

**Figure A2.**
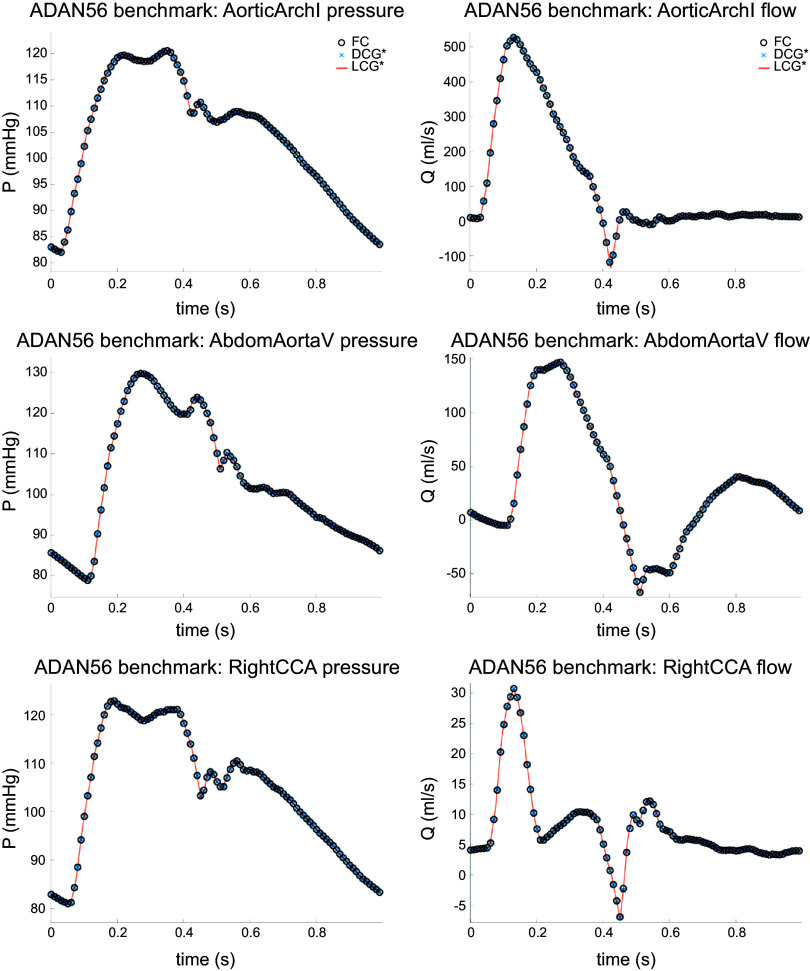
A 77-segment (ADAN56) benchmark. Pressure (*left*) and flow rate (*right*) at the midpoint of (*top* to *bottom*) the aortic arch (AorticArchI), abdominal aorta (AbdomAortaV), and right common carotid artery (RightCCA) simulated by Fourier continuation (FC), discontinuous Galerkin (DCG) (37), and locally conservative Galerkin (LCG) (64) methods are shown. Lines are plotted at a resolution of Δ*t* = 10^−3^ s, and markers are plotted every 10 such time steps (10^−2^ s) for easier visualization. *Provided in Boileau et al. (37).

## APPENDIX

### The Numerical Model

The numerical methodology for solving the partial differential equation (PDE) system of *Eqs. 1* and *2* is based on a Fourier continuation (FC) approach that has been introduced previously for 1-D arterial wave propagation (29). Both implicit and explicit FC-based PDE solvers have been successfully constructed and used for a variety of physical problems, including those governed by advection-diffusion equations, Navier–Cauchy elastodynamics equations, Navier–Stokes fluid equations, and fluid-structure equations (28, 29, 38, 62, 63).

Validations of the complete numerical solver have been conducted using the benchmark problems proposed by Boileau et al. (37). Figure A1 presents pressure and flow solutions of a single-segment common carotid artery (with Windkessel) benchmark using both FC as well as commonly used discontinuous Galerkin (DCG) (37) and locally conservative Galerkin (LCG) methods (64). Figure A2 additionally presents corresponding FC-, DCG-, and LCG-simulated pressure and flow at midpoints of various vascular segments from a benchmark problem (37) on a 77-segment open-loop model (that considers 56 major arteries). Parameters for these benchmarks can be found in the respective source (37). For both problems, Figs. A1 and A2 demonstrate excellent correspondence between the solutions generated by the FC solver employed in this work and those generated by other numerical schemes.

### Aortic Wave Intensity Pattern

Figure A3 presents calculated aortic WI patterns from simulations at different contractility values. These patterns are presented for the normal heart rate (75 beats/min) and the baseline aortic stiffness. Results indicate a distinct midsystolic forward traveling front (which is a function of contractility) in both carotid and aortic WI patterns, with it being more pronounced in the carotid, as demonstrated in Fig. 8.

### Statistical Analysis

The summary of the Shapiro–Wilk test to check the normality of each regression’s residuals is presented in Table A1.

**Table A1.** Summary of Shapiro–Wilk test to check normality

Model	*P* Value
Model with the following dependent variables	
CPI	0.471
CPP	0.957
CBF	0.979

CBF, cerebral blood flow; CPI, carotid pulsatility index; CPP, carotid pulsatile power.

The regression model outcomes for three dependent variables, CPI, CPP, and CBF, are presented in Tables A2–A4, respectively. Independent variables in this study are LV contractility, aortic PWV, and heart rate. All independent variables are incorporated in the models as categorical variables. The minimum baseline for the heart rate is 30 beats/min, for the contractility is 0.6 mmHg/mL and the aortic PWV is $c_{0}$.

**Table A2.** Summary of regression statistics for CPI as a dependent variable

Variable	Estimate	Robust Standard Error (HC3)	*t* Statistics	*P* Value
Intercept	4.176	0.332	12.563	<0.001
Heart rate, beats/min				
50	0.205	0.267	0.7673	0.444
75	0.348	0.257	1.353	0.178
100	1.438	0.262	5.497	<0.001
125	2.954	0.289	10.210	<0.001
180	4.039	0.321	12.581	<0.001
Contractility, mmHg/mL				
1.2	0.223	0.262	0.851	0.397
1.8	0.350	0.238	1.469	0.144
2.5	0.578	0.232	2.494	0.013
3.5	0.936	0.262	3.570	<0.001
5.0	1.992	0.304	6.549	<0.001
PWV				
1.5 c_0_	2.184	0.180	12.150	<0.001
2.0 c_0_	3.333	0.190	17.554	<0.001
3.0 c_0_	4.211	0.210	20.073	<0.001

CPI, carotid pulsatility index; PWV, pulse wave velocity.

**Table A3.** Summary of regression statistics for CPP as a dependent variable

Variable	Estimate	Robust Standard Error (HC3)	*t* Statistics	*P* Value
Intercept	7.642	0.4383	17.436	<0.001
Heart rate, beats/min				
50	−5.2815	0.3392	−15.569	<0.001
75	−6.9915	0.3628	−19.273	<0.001
100	−6.5587	0.3538	−18.537	<0.001
125	−5.172	0.332	−15.584	<0.001
180	−2.034	0.4437	−4.584	<0.001
Contractility, mmHg/mL				
1.2	0.2442	0.334	0.731	0.446
1.8	0.483	0.320	1.51	0.133
2.5	0.604	0.316	1.909	0.058
3.5	1.195	0.330	3.625	<0.001
5.0	1.9974	0.3776	5.289	<0.001
PWV				
1.5 c_0_	2.785	0.241	11.547	<0.001
2.0 c_0_	4.292	0.263	16.319	<0.001
3.0 c_0_	5.582	0.309	18.042	<0.001

CPP, carotid pulsatile power; PWV, pulse wave velocity.

**Table A4.** Summary of regression statistics for CBF as a dependent variable

Variable	Estimate	Robust Standard Error (HC3)	*t* Statistics	*P* Value
Intercept	0.709	0.005	149.144	<0.001
Heart rate, beats/min				
100	0.003	0.003	0.936	0.352
125	0.016	0.004	3.921	<0.001
180	0.008	0.003	2.434	0.017
Contractility, mmHg/mL				
1.2	0.006	0.003	1.879	0.064
1.8	0.013	0.004	3.394	0.001
2.5	0.012	0.003	3.646	<0.001
3.5	0.032	0.004	7.757	<0.001
5.0	0.0288	0.004	6.541	<0.001
PWV c_0_				
1.5	−0.017	0.003	−5.316	<0.001
2.0	−0.025	0.003	−7.739	<0.001
3.0	−0.032	0.003	−9.144	<0.001

CBF, cerebral blood flow; PWV, pulse wave velocity.
